# Cardiac baroreflex is already blunted in eight weeks old spontaneously hypertensive rats

**DOI:** 10.1186/1755-7682-3-2

**Published:** 2010-01-27

**Authors:** José R Cisternas, Vitor E Valenti, Thales B Alves, Celso Ferreira, Márcio Petenusso, João R Breda, Adilson C Pires, Nadir Tassi, Luiz Carlos de Abreu

**Affiliations:** 1 Department of Morphology and Physiology, School of Medicine of ABC, Av. Príncipe de Gales, 821, Santo André 09060-650, Brazil; 2 Department of Clinical Medicine, Cardiology Division, School of Medicine of ABC, Av. Príncipe de Gales, 821, Santo André 09060-650, Brazil; 3 Department of Medicine, Cardiology Division Federal University of São Paulo (UNIFESP), Rua Napoleão de Barros, 715 Térreo, São Paulo 04039-032, Brazil; 4 Department of Physiology, Federal University of São Paulo (UNIFESP), Rua Napoleão de Barros, 715 Térreo, São Paulo 04039-032, Brazil; 5 Cardiovascular Surgery Division, School of Medicine of ABC, Av. Príncipe de Gales, 821, Santo André 09060-650, Brazil; 6 Speech Pathology and Audiology Department, Faculty of Philosophy and Sciences, Paulista State University (UNESP), Av. Hygino Muzzi Filho, 737, Campus Universitário, Marília 17525-900, Brazil

## Abstract

**Background:**

The literature did not evidence yet with which age spontaneously hypertensive rats (SHR) start to present baroreflex reduction. We endeavored to evaluate the baroreflex function in eight-week-old SHR.

**Methods:**

Male Wistar Kyoto (WKY) normotensive rats and SHR aged eight weeks were studied. Baroreflex was calculated as the variation of heart rate (HR) divided by the mean arterial pressure (MAP) variation (ΔHR/ΔMAP) tested with a depressor dose of sodium nitroprusside (SNP, 50 μg/kg) and with a pressor dose of phenylephrine (PHE, 8 μg/kg) in the right femoral venous approach through an inserted cannula in the animals. Significant differences for p < 0.05.

**Results:**

Baseline MAP (p < 0.0001) and HR (p = 0.0028) was higher in SHR. Bradycardic peak was attenuated in SHR (p < 0.0001), baroreflex gain tested with PHE was also reduced in the SHR group (p = 0.0012). PHE-induced increase in MAP was increased in WKY compared to SHR (p = 0.039). Bradycardic reflex responses to intravenous PHE was decreased in SHR (p < 0.0001).

**Conclusion:**

Eight weeks old SHR already presents impairment of the parasympathetic component of baroreflex.

## Introduction

Several factors (neural, humoral, myogenic) are involved in the onset of hypertension and different animal models have been used to study this pathology, such as the renal hypertension model, the DOCA-salt hypertension model, the neurogenic hypertension model and the genetic model of hypertension in spontaneously hypertensive rats (SHR) [1]. SHR is a suitable model to study hypertension development as it is similar to humans with essential hypertension. These similarities include a genetic predisposition to high blood pressure with no specific etiology, increased total peripheral resistance without volume expansion and similar responses to drug treatment [1]. Moreover, cardiac hypertrophy, a stress model of heart disease [2-4] is another feature of SHR [5].

In cardiovascular physiology, the baroreflex or baroreceptor reflex is one of the body's homeostatic mechanisms to maintain blood pressure. It provides a negative feedback loop in which the elevated blood pressure reflexively causes blood pressure to decrease; in contrast, the decreased blood pressure depresses the baroreflex, causing blood pressure to rise. The system relies on specialized neurons (baroreceptors) in the aortic arch, carotid sinuses and elsewhere to monitor changes in blood pressure and relay them to the brainstem. Subsequent changes in blood pressure are mediated by the autonomic nervous system [6]. Previous studies related to the development of young SHR baroreflex function have yielded conflicting results, when compared to normotensive control rats (Wistar-Kyoto - WKY) [7]. A study of Lundin et al [8] showed that 15 weeks old present reduced baroreflex function. Furthermore, a recent investigation evidenced that 13 weeks old SHR already present impaired baroceptor reflex with respect to the parasympathetic component [9]. A precise knowledge of early development damage to the baroreflex function is essential to understand hypertension as a disease process [10,11]. Therefore, in this study we investigated cardiac baroreflex in eight weeks old SHR.

## Methods

### Animals

Eight weeks old SHR (n = 19) and WKY (n = 35) rats were kept in the Animal Care Unit of our University. We used higher number of WKY rats in order to have trustful control values. Rats were housed individually in plastic cages under standard laboratory conditions. They were kept under a 12 h light/dark cycle (lights on at 07:00 h) and had free access to food and water. The Institution's Animal Ethics Committee authorized housing conditions and experimental procedures.

### Surgical Preparation

One day before the experiments the rats were anesthetized with ketamine (50 mg/kg i.p.) and xilazine (10 mg/kg i.m.) and a catheter was inserted into the abdominal aorta through the femoral artery for blood pressure and heart rate recording. Catheters were made of 4 cm segments of PE-10 polyethylene (Clay Adams, USA) heat bound to a 13 cm segment of PE-50 (Clay Adams, USA). The catheters were tunneled under the skin and exteriorized at the animal's dorsum [9,12].

### Arterial pressure and heart rate recording

After surgery, the animals were kept in individual cages used in the transport to the recording room. Approximately 24 hours after the surgery, animals were allowed 20 min to adapt to the conditions of the experimental room such as sound and illumination before starting blood pressure and heart rate recording. The experimental room was acoustically isolated and had constant background noise produced by an air exhauster. At least another 15 min period was allowed before beginning experiments. Pulsatile arterial pressure (PAP) of freely moving animals was recorded using an HP-7754A preamplifier (Hewlett Packard, USA) and an acquisition board (MP100A, Biopac Systems Inc, USA) connected to a computer. Mean arterial pressure (MAP) and heart rate (HR) values were derived from the PAP recordings and processed on-line [9,12].

### Baroreflex Test

The baroreflex was tested with a pressor dose of 0.1 mL phenylephrine (PHE-bolus-8 μg/kg IV; Sigma Chemical) and depressor doses of 0.1 mL sodium nitroprusside (SNP-bolus-50 μg/kg IV; RBI) [9,12].

### Baroreflex evaluation

The baroreflex gain was calculated as the derivation of HR in function of the MAP variation (ΔHR/ΔMAP). We also analyzed bradycardic and tachycardic peak and HR range (the difference between bradycardic and tachycardic peak) [9,12].

### Statistical Analysis

Values are reported as the means ± standard error of means. HR, MAP, ΔHR, ΔMAP and ΔHR/ΔMAP were compared between WKY and SHR. After the distributions were evaluated through the Kolmogorov normality test, the unpaired *Student's T *test was used to verify differences between normal distributions and the Mann-Whitney test was used to assess differences between non-parametric distributions. Differences were considered significant when the probability of a Type I error was less than 5% (*p *< 0.05).

## Results

As shown in Table 1, we observed significant difference between SHR and WKY control groups regarding baseline MAP and HR. Furthermore, the values of bradycardic peak in SHR were higher than WKY rats. Hence, their bradycardic peak caused by MAP increase was attenuated. Whereas tachycardic peak was significantly increased in the same group, however, there was no significant difference with respect to HR range. Baroreflex gain tested with PHE was also significantly reduced in the SHR group. On the other hand, we did not observed significant alterations regarding baroreflex gain tested with SNP.

**Table 1:** Baseline level of mean arterial pressure (MAP) and heart rate (HR), bradycardic and tachycardic peak, HR range and baroreflex gain (BG) in SHR (n = 19) and WKY (n = 35) rats

*Variable*	*WKY*	*SHR*	*P Value*
***MAP (mmHg)***	113.4 ± 1.66	162.89 ± 2.7	< 0.0001
***HR (bpm)***	313.34 ± 7.1	350 ± 8.9	0.0028
***Bradycardic Peak (bpm)***	242.5 ± 7.2	313.47 ± 9.4	< 0.0001
***Tachycardic Peak (bpm)***	444.56 ± 8.4	490 ± 9	0.0013
***HR range (bpm)***	198.03 ± 8.12	184.11 ± 13.35	0.3493
***BG (bpm × mmHg^-1^) PHE***	-1.3623 ± 0.1	-0.851 ± 0.09	0.0012
***BG (bpm × mmHg^-1^) NaNP***	-2.57 ± 0.18	-2.5 ± 0.26	0.837

Unpaired Student T test.

**Figure 1:**
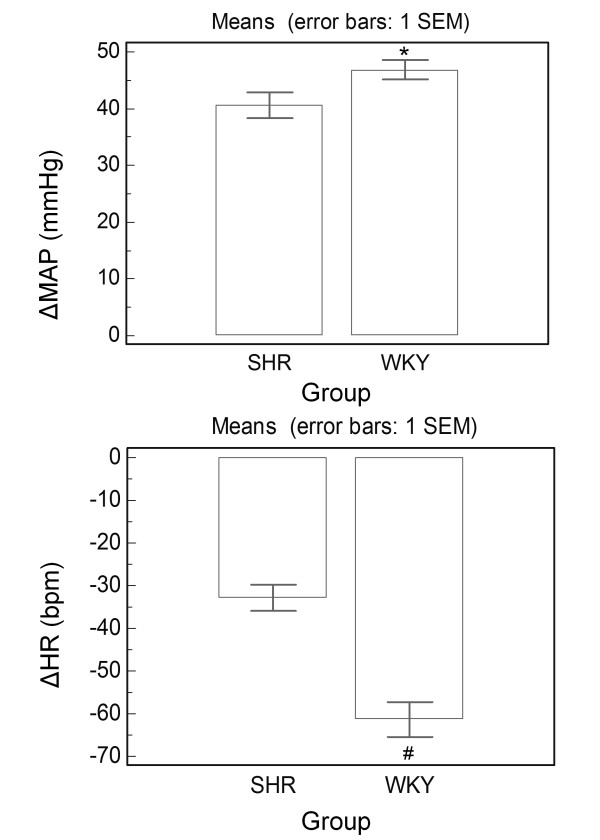
**Increase in mean arterial pressure (MAP, mmHg) and decrease in heart rate (HR, bpm) in response to phenylephrine (PHE, 8 μg/kg i.v.) in SHR (n = 19) and WKY (n = 35) rats**. *p = 0.039; ^#^p < 0.0001: Different of SHR. Unpaired Student T test.

**Figure 2:**
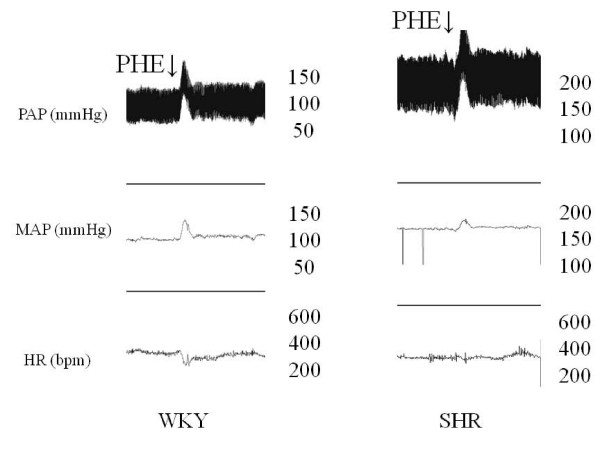
**Recordings from one WKY and one SHR illustrating reflex bradycardia (top) in response to blood pressure increases**. Infusions were given in bolus. MAP, mean arterial pressure; PAP, pulsatile arterial pressure; HR, heart rate; PHE: phenylephrine.

PHE-induced increase in MAP was slightly but significantly increased in WKY rats compared to the SHR group (p = 0.039). Moreover, bradycardic reflex responses to intravenous PHE was significantly decreased in SHR (p < 0.0001) (Figure 1). Figure 2 presents representative recordings obtained during baroreflex testing with PHE in conscious WKY and SHR, showing expressive difference between WKY and SHR groups in relation to PHE-induced increase in arterial pressure. The reflex bradycardia in response to PHE was significantly reduced in SHR.

**Figure 3:**
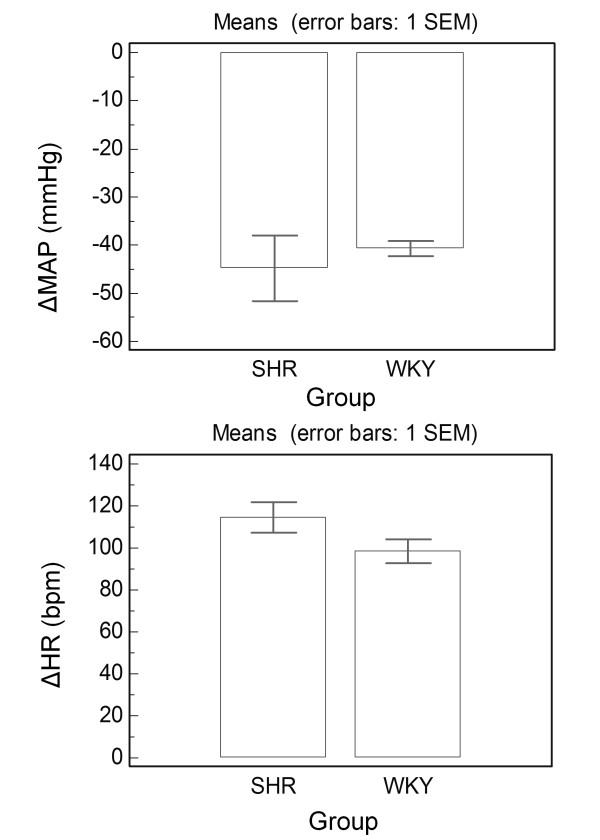
**Decrease in mean arterial pressure (MAP, mmHg) and decrease in heart rate (HR, bpm) in response to sodium nitroprusside (SNP, 50 μg/kg i.v.) in SHR (n = 19) and WKY (n = 35) rats**. Unpaired Student T test.

**Figure 4:**
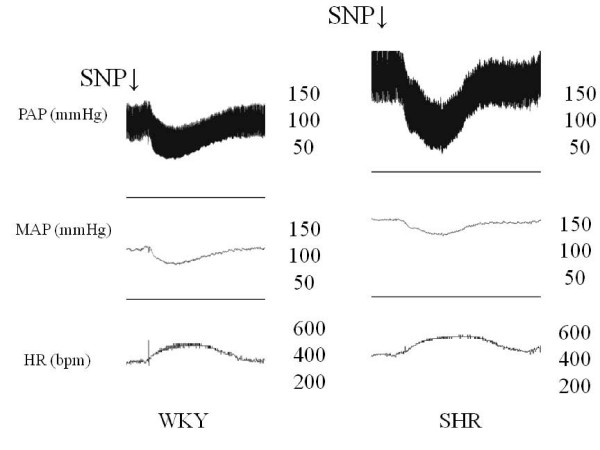
**Recordings from one control WKY and one SHR illustrating reflex tachycardia (top) in response to blood pressure decreases**. Infusions were given in bolus. MAP, mean arterial pressure; PAP, pulsatile arterial pressure; HR, heart rate; SNP: sodium nitroprusside.

Intravenous injections of SNP produced a vasodepressor response, which was similar in both groups (p = 0.46). Moreover, tachycardic reflex in response to SNP-induce decrease in MAP tended to be impaired in SHR group but it did not reach statistical significance (p = 0.1) (Figure 3). We observe in Figure 4 representative recordings obtained during baroreflex testing with SNP in conscious WKY and SHR, and it is noted no expressive difference between the groups. The tachycardic reflex in response to SNP-induced decrease in arterial pressure was similar in both groups.

## Discussion

Our investigation was undertaken to evaluate baroreflex function in eight weeks old SHR tested with SNP and PHE and to verify whether there is any difference between this study model and WKY rats at the same age. Our findings demonstrate that at this age baseline MAP and HR are already increased in SHR, which is supported by the literature [9,13]. In addition, pressor responses to PHE were increased in WKY whereas bradycardic reflex was reduced in SHR. Bradycardic peak, HR range and the parasympathetic component of the baroreflex gain were attenuated in SHR. On the other hand, no significant differences were noted in relation to SNP-induced decrease in MAP, tachycardic reflex and baroreflex gain tested with SNP.

The bradycardic peak is an index of maximal parasympathetic response to PHE-induced increase in blood pressure; the tachycardic peak represents the maximal sympathetic response to SNP-induced decrease in arterial pressure; the HR range index represents the difference between the upper and lower HR peak and the derivation of HR in function of MAP variation is an index of baroreflex gain [14]. We reported that the maximal parasympathetic activity is already attenuated in eight weeks old SHR compared to age-matched control WKY rats. Our findings provide important evidence for the recent hypothesis that young SHR already present reduced parasympathetic function.

Our findings indicate no significant impairment of the sympathetic component of baroreflex between eight weeks old SHR and WKY rats at the same age. Great attention has focused on the role of the sympathetic activity regarding the onset of hypertension in SHR. Previous studies have shown that there is elevated sympathetic drive to the vessels in adult SHR and have suggested that this is relevant in the maintenance of increased blood pressure [8,15]. It is possible that this elevation in sympathetic output is not primarily a consequence of changes in either baroceptor reflex [15] or chemoreflex function but rather is a product of a modification of the central neural circuitry involved in generating the sympathetic output [16]. In view of the above considerations, we expected the absence of difference between eight weeks old SHR and age-matched WKY rats with respect to tachycardic peak, tachycardic reflex and sympathetic baroreflex gain.

The mechanisms that cause the reduction of the baroreflex function in SHR are not completely understood [1]. Some studies investigated issues associated to afferent limb, for example, one investigation demonstrated that the carotid body in the adult SHR is significantly larger than in normotensive rats [17,18], whereas other studies indicated that the decreased baroreflex function in SHR is due to impaired levels of norephinephrine, epinephrine and dopamine in the carotid body [19,20]. Other researchers studied the central nervous system [19-21]. It was evidenced that impaired levels of norephinephrine, epinephrine and dopamine into the medulla oblongata areas that regulate the cardiovascular system [19-21] may be involved in baroreflex derangement in SHR. Waki et al [22] have shown that endogenous nitric oxide synthase activity in the medulla oblongata of SHR is increased when compared to WKY; it plays a major role in the preservation of the hypertension and decreases the cardiac baroreceptor reflex gain, which are features of this animal model. Furthermore, peripherical mechanisms are also associated to attenuated baroreflex development in this strain, there have been reports that AT1 (angiotensin) receptor densities are increased in SHR, compared to the levels found in normotensive control rats [23]. Rossi et al [23] indicated that endogenous endothelin receptor mechanisms are involved in the hypertensive state observed in SHR. Moreover, recent investigations described the importance of oxidative stress [24] and the small GTPase rho-quinase [25] during baroreflex function development in SHR. Bertagnoli et al [24] suggested that exercise training reduces oxidative stress, which is associated to an improvement in baroreflex sensitivity in SHR. Earlier studies that described a significant difference in blood pressure between WKY and SHR at 3 weeks of age [26] or at birth [27] were based on the assessment of a few animals. However, the age at which the baroreflex function starts to decrease in SHR has yet to be demonstrated.

PHE-induced increase in MAP was significantly enhanced in WKY group compared to SHR group and bradycardic reflex response to increase in arterial pressure was also significantly increased in WKY rats. We believe that PHE-induced increase in MAP was reduced in SHR due their high blood pressure, which disable them to present high variation of MAP. The reduced bradycardic reflex response to increase in arterial pressure in SHR is explained by their reduced parasympathetic activity, which decrease the parasympathetic component of baroreflex [7,16,28]. However, it is not known yet with which age SHR begins to present prevalence of sympathetic activity. A recent study suggests that the sympathetic activity starts to increase at newborn stage [16]. On the other hand, no previous investigation demonstrated when SHR begin to show attenuated bradycardic reflex.

SNP-induced decrease in MAP and tachycardic reflex was similar between WKY and SHR groups. Those parameters were used to calculate the sympathetic baroreflex gain (baroreflex deactivation, ΔHR/ΔMAP). Thus, the sympathetic baroreflex gain was also similar between the both groups.

We report that SHR presented impairment of the parasympathetic component of baroreflex function, associated with preservation of sympathetic component. It is well described that sympathetic activity is increased during stress conditions [2-4,29] and SHR is also a model to study hyperactivity and stress disorders [30,31]. The imbalance between sympathetic and parasympathetic components of the baroreflex in SHR is associated to their sympathetic hyperactivity. Hence, this imbalance collaborates to hypertension development in this strain [24,25]. Our findings suggest that at eight weeks old this discrepancy between sympathetic and parasympathetic baroreflex gain in SHR probably cooperates to keep increasing arterial pressure.

These data present relevant information, since currently baroceptor reflex is largely studied in different models and strain of rats aiming to prevent hypertension development in human [9-11], due the fact that reduced baroreflex function is indicative of cardiovascular disease [9,14,15]. We recognize the limitations of our analysis in that we are unable to provide a full baroreceptor reflex function curve. However, the baroreflex gain values obtained here are of physiological relevance, because they fall around the operating point of this reflex in an unrestrained conscious rat [12,25],

In conclusion, our investigation indicates that eight weeks old SHR already presents impairment of the parasympathetic component of baroreflex function, while no difference was observed regarding the sympathetic component.

## Competing interests

The authors declare that they have no competing interests.

## Authors' contributions

JRC, VEV, LCA, CF, NT, JRB, ACP and TBA performed the experimental procedures and helped to write the manuscript. VEV, MP and LCA carried out the statistical analysis and participated in design the manuscript. All authors read and approved the final manuscript.
